# Concurrent, but not sequential, PD-1 blockade with a DNA vaccine elicits anti-tumor responses in patients with metastatic, castration-resistant prostate cancer

**DOI:** 10.18632/oncotarget.25387

**Published:** 2018-05-22

**Authors:** Douglas G. McNeel, Jens C. Eickhoff, Ellen Wargowski, Christopher Zahm, Mary Jane Staab, Jane Straus, Glenn Liu

**Affiliations:** ^1^ University of Wisconsin Carbone Comprehensive Cancer Center, Madison, WI 53705, USA; ^2^ Department of Biostatistics, University of Wisconsin, Madison, WI 53792, USA

**Keywords:** DNA vaccine, prostate cancer, prostatic acid phosphatase, pembrolizumab, PD-1

## Abstract

T-cell checkpoint inhibitors have demonstrated dramatic clinical activity against multiple cancer types, however little activity in patients with prostate cancer. Conversely, an anti-tumor vaccine was approved for the treatment of prostate cancer, having demonstrated an improvement in overall survival, despite few objective disease responses. In murine studies, we found that PD-1 expression on CD8+ T cells increased following anti-tumor vaccination, and that PD-1/PD-L1 blockade at the time of immunization elicited greater anti-tumor responses. Based on these data we initiated a pilot trial evaluating the immunological and clinical efficacy of a DNA encoding prostatic acid phosphatase (PAP) when delivered in combination with pembrolizumab. 26 patients were treated for 12 weeks with vaccine and received pembrolizumab either during this time or during the subsequent 12 weeks. Adverse events included grade 2 and 3 fatigue, diarrhea, thyroid dysfunction, and hepatitis. Median time to radiographic progression was not different between study arms. 8/13 (62%) of patients treated concurrently, and 1/12 (8%, p=0.01) of patients treated sequentially, experienced PSA declines from baseline. Of these, two were over 50% and one was a complete PSA response. No confirmed CR or PR were observed, however 4/5 patients treated concurrently had measurable decreases in tumor volume at 12 weeks. PSA declines were associated with the development of PAP-specific Th1-biased T cell immunity and CD8+ T cell infiltration in metastatic tumor biopsy specimens. These data are the first report of a clinical trial demonstrating that the efficacy of an anti-tumor vaccine can be augmented by concurrent PD-1 blockade.

## INTRODUCTION

Prostate cancer is the third leading cause of cancer-related death in men in the United States [1]. Metastatic, castration-resistant prostate cancer (mCRPC) is the lethal form of the disease, accounting for the majority of deaths due to prostate cancer. Over the last 10 years, several therapies have been approved by FDA based on their ability to prolong overall survival in this population of patients. These agents include docetaxel [2, 3], cabazitaxel [4], sipuleucel-T [5], abiraterone [6], enzalutamide [7], and radium-223 [8]. Notwithstanding, the median survival benefit of each of these agents is on the order of 3 to 4 months, demonstrating that therapies with greater benefit are urgently needed.

T-cell checkpoint inhibitors, such as antibodies targeting CTLA-4 or PD-1/PD-L1, have demonstrated remarkable activity for some cancers, notably melanoma, with profound and enduring clinical responses [9, 10]. To date, however, while some clinical responses have been observed in patients treated with pembrolizumab in combination with enzalutamide, little objective benefit has been observed with these therapies employed as monotherapies for patients with prostate cancer [11–13]. On the other hand, a vaccine aimed at increasing tumor-specific immunity has demonstrated benefit in terms of prolonged overall survival. Sipuleucel-T, a vaccine targeting prostatic acid phosphatase (PAP), is currently the only anti-tumor vaccine approved by FDA for the treatment of cancer, and was approved on the basis of improved overall survival [5]. However, favorable radiographic and PSA changes with sipuleucel-T treatment are rare.

We have previously reported that a DNA encoding PAP (pTVG-HP) could be safely administered to patients with early PSA-recurrent prostate cancer and elicit/augment PAP-specific Th1-biased T cells [14, 15]. While PSA declines following treatment were rare, favorable changes in PSA doubling time were observed, and these changes were associated with the development of Th1-biased immunity [15, 16]. In preclinical studies, we have explored different approaches to increase the immune response from DNA immunization. We found that efforts to increase the magnitude of immune response by encoding epitopes with greater MHC class I affinity led to inferior anti-tumor efficacy in tumor-bearing mice [17]. This was due to the expression of PD-1 on antigen-specific CD8+ T cells elicited with immunization, and blockade of PD-1 or PD-L1 at the time of T-cell activation with immunization led to superior anti-tumor efficacy [17, 18]. In other preclinical studies, we found that patients previously immunized with either pTVG-HP or sipuleucel-T developed PD-1-regulated immune responses, and that circulating tumor cells increased expression of PD-L1 after immunization [19].

On the basis of these data, we initiated a pilot clinical trial evaluating a DNA vaccine delivered concurrently or sequentially with PD-1 blockade, using pembrolizumab, in patients with mCRPC. The goal was to determine whether PD-1 blockade at the time of T-cell activation with vaccination (during T-cell activation) was superior to blockade after vaccination (following activation that potentially induces a PD-1-regulated immune response). We report here the safety, clinical, and correlative immunological data from this trial. To our knowledge, this is the first report of a clinical trial using a PD-1 antagonist with a tumor-specific DNA vaccine employed as a T-cell activating agent.

## RESULTS

### Patient population and course of study

**Figure 1:**
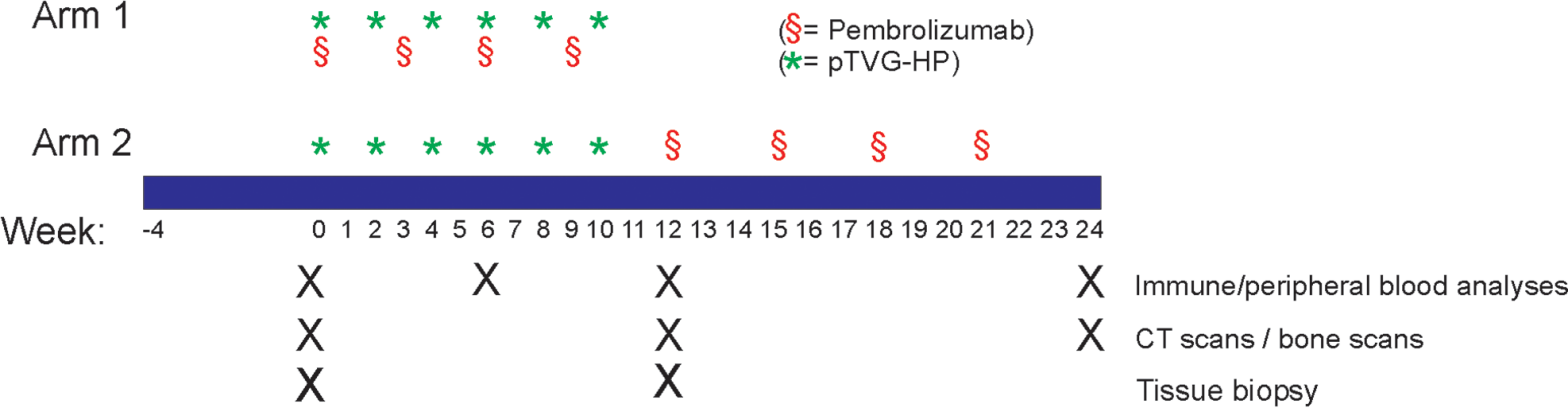
Schema Shown is the treatment schema.

26 patients with progressive, mCRPC were enrolled in this trial between August 2015 and May 2017 at the University of Wisconsin Carbone Cancer Center. The general schema for the treatment is shown in Figure 1. Demographic information and prior treatments are indicated in Table 1. The median age of participants was 73 years (range 56-85 years). Over the course of treatment, no grade 4 adverse events were observed. Grade 3 events included one episode each of fatigue, adrenal insufficiency, diarrhea, hepatitis, pancreatitis, and syncope. Grade 2 events believed at least possibly related to study treatment and occurring in over 5% of subjects overall included fatigue, diarrhea, hypothyroidism, hyperthyroidism, and pain (Table 2). All these events were suspected to be related to pembrolizumab. A patient with grade 3 diarrhea (Arm 2) died within 30 days of coming off trial, and 46 days after his last treatment with pembrolizumab, due to multi-organ failure. While that patient had progressive disease, he declined further therapy or evaluation and hence attribution to the study agents could not be excluded. All but two patients completed study treatments; one in each study arm (20004 and 10007) had evidence of progressive disease within the first month and consequently came off study early. Blood was obtained at post-treatment time points for immune analysis for one of these patients (10007), but was not available for the other. One patient in Arm 2 (20002), while meeting eligibility criteria, experienced a delayed bicalutamide withdrawal response, with a decreased PSA at day 1 of treatment, and hence was not evaluable for PSA response.

**Table 1:** Demographics

	Total (N=26)	Arm 1 (N=13)	Arm 2 (N=13)
Age (median and range, years)	73 (56-85)	69 (62-83)	75 (56-85)
Race:			
Caucasian	26 (100%)	13 (100%)	13 (100%)
Initial Gleason Score:			
Unknown			
< 7	2 (8%)	0 (0%)	2 (15%)
7	5 (19%)	3 (23%)	2 (15%)
8	5 (19%)	3 (23%)	2 (15%)
9	14 (54%)	7 (54%)	7 (54%)
Prior treatments:			
Prostatectomy	11 (42%)	6 (46%)	5 (38%)
Radiation therapy (primary, salvage, or palliative)	14 (54%)	6 (46%)	8 (62%)
LHRH (or orchiectomy) +/- bicalutamide	26 (100%)	13 (100%)	13 (100%)
Chemotherapy (docetaxel or cabazitaxel)	5 (19%)	2 (15%)	3 (23%)
Abiraterone	3 (12%)	1 (8%)	2 (15%)
Enzalutamide	3 (12%)	1 (8%)	2 (15%)
Other investigational agents	11 (42%)	5 (38%)	6 (46%)
Pre-treatment:			
Baseline serum PSA, ng/mL (median, range)	24 (3-165)	25 (3-150)	24 (3-165)

**Table 2:** Adverse events

	Grade 2		Grade 3		Grade 4		Grade 5	
	Arm 1	Arm 2	Arm 1	Arm 2	Arm 1	Arm 2	Arm 1	Arm 2
General/Constitutional								
Fatigue	3 (23%)	3 (23%)	1 (4%)					
Weight loss		1 (4%)						
Endocrine								
Adrenal insufficiency				1 (4%)				
Hyperthyroidism	1 (8%)	1 (8%)						
Hypothyroidism	2 (15%)	2 (15%)						
Gastrointestinal								
Abdominal pain		1 (4%)						
Constipation		1 (4%)						
Diarrhea	1 (4%)			1 (4%)				
GE reflux		1 (4%)						
Hepatitis				1 (4%)				
Nausea	1 (4%)							
Pancreatitis				1 (4%)				
Laboratory investigations								
Increased ALT		1 (4%)						
Increased AST	1 (4%)			1 (4%)				
Increased alk phos		1 (4%)						
Metabolism/Nutrition								
Hyperglycemia		1 (4%)						
Musculoskeletal								
Arthralgia		1 (4%)						
Back pain		1 (4%)						
Nervous system								
Syncope			1 (4%)					
Reproductive system								
Scrotal pain		1 (4%)						
Vascular disorders								
Hot flashes	1 (4%)							
Multi-organ failure								1 (4%)

All adverse events by grade > grade 1 that were believed to be at least possibly related to treatment are shown. The numbers represent the number of patients (out of 22) experiencing a particular event at any point during the 3- to 6-month treatment period, with the highest grade reported for any single individual.

### Immunological response

**Figure 2:**
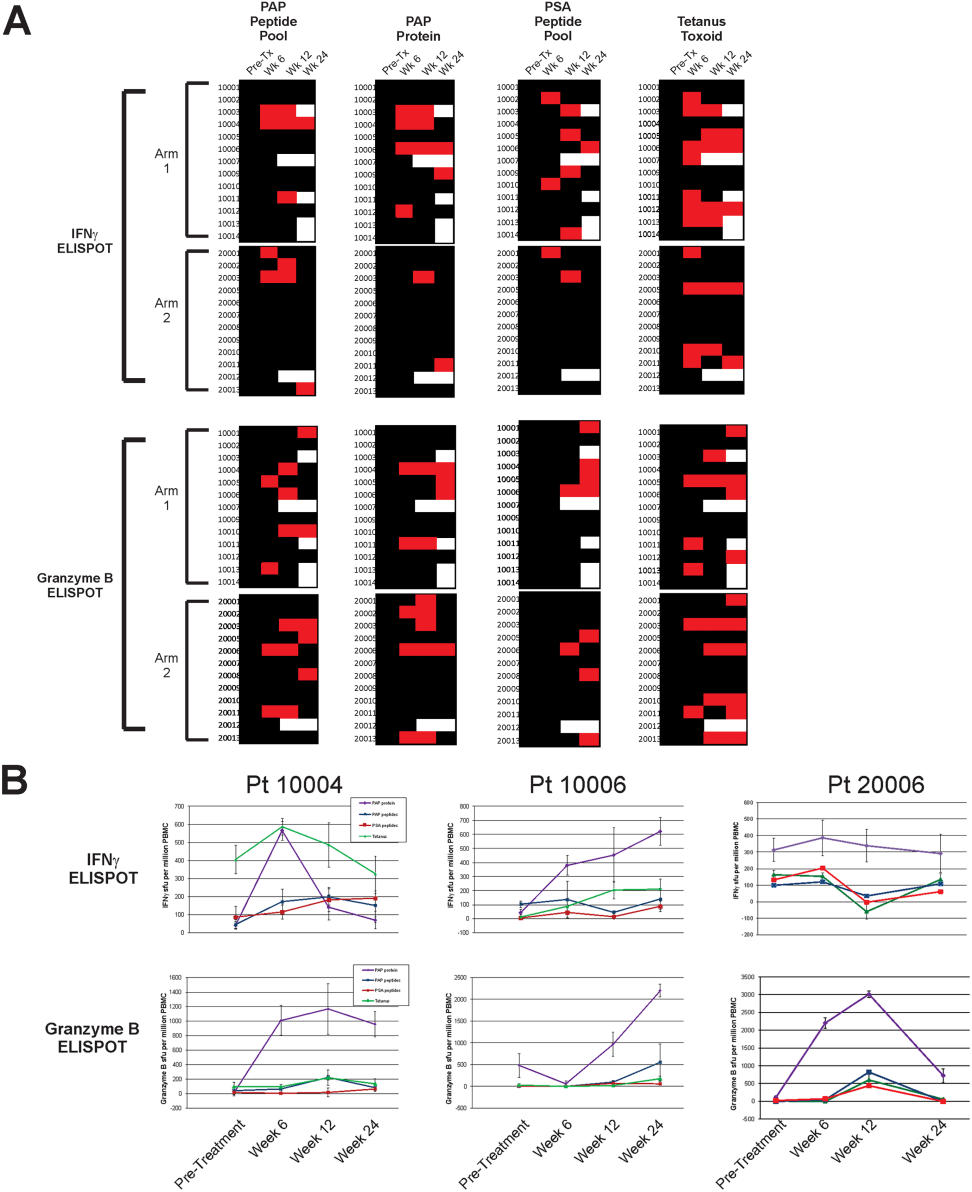
Immunological response - IFNγ and Granzyme B ELISPOT Peripheral blood mononuclear cells were collected from all subjects at baseline, 6 weeks, 12 weeks, and 24 weeks and evaluated for antigen-specific IFNγ or granzyme B secretion by ELISPOT. **(A)** Shown are immune responses to PAP protein or peptide library, PSA peptide library (non-specific control), and tetanus (positive control) for patients grouped by study arm. A positive antigen-specific response was defined as a statistically significant response (compared with media control) that was at least 3-fold over the baseline value and with a frequency of at least 1/100,000 cells. Red squares indicate positive responses, black indicates no response, and white indicates no data. **(B)** Shown are individual ELISPOT data for the three individuals (two from Arm 1 – 10004 and 10006, and one from Arm 2 – 20006) who exhibited PAP-specific immunity at all post-treatment time points in panel A.

Patients were evaluated prior to treatment, and at weeks 6, 12 and 24 for evidence of T-cell immunity to the PAP target antigen (using either PAP protein or a pool of peptides spanning the amino acid sequence of PAP) by ELISPOT. A significant increase in PAP-specific IFNγ-secreting T cells was detected in multiple subjects treated on either study arm (Figure 2A). 11/25 (44%, 95% CI: 27-63%) individuals had evidence of immunity, with significant increases in PAP-specific T cells detectable at one or more post-treatment time points. Cytolytic type responses specific for PAP, secreting granzyme B, were also detectable; 15/25 (60%, 95% CI: 41-77%) of individuals had evidence of PAP-specific granzyme B-secreting cells detected at one or more post-treatment time point. IFNγ- and/or granzyme B-secreting T cell responses to PAP were detectable in 10/25 (40%, 95% CI: 23-59%) of individuals at more than one post-treatment time point. The frequency and magnitude of response were not different between study arms. T-cell immunity to tetanus was detectable at more than one post-treatment time point in 11/25 (44%, 95% CI: 27-63%) patients, as expected, given that patients received a tetanus immunization prior to study treatment to provide a positive control. Responses to PSA were evaluated as a non-target prostate cancer antigen. As expected, while responses were detectable at discreet time points, IFNγ- and/or granzyme B-secreting T cell responses were only detectable in 1/25 patients (4%, 95% CI: 0-20%) at more than one post-treatment time point. Three patients, two in Arm 1 and one in Arm 2, had evidence of PAP-specific IFNγ- or granzyme B-secreting T cells at all post-treatment time points (Figure 2B).

### Clinical effects

**Figure 3:**
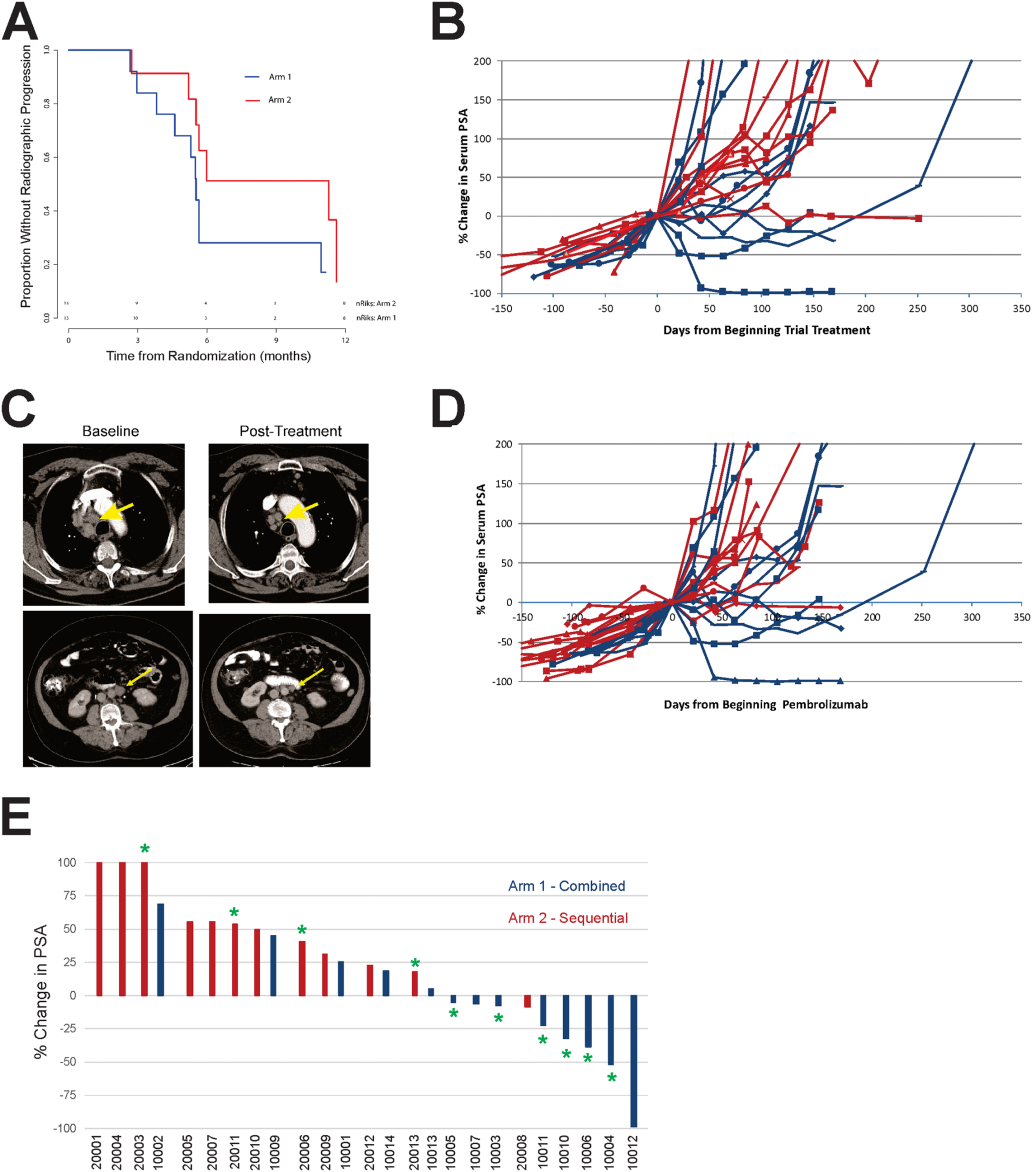
Clinical effects **(A)** Kaplan-Meier plot of time to radiographic progression by study arm. Time to radiographic regression was defined as the time from randomization to the date of documented radiographic progression or last available follow-up date. **(B)** Serum PSA values were collected from all individuals prior to treatment and over the course of treatment. Percent changes in serum PSA values were evaluated from day 1 of study. Blue lines show individual patients treated in Arm 1, and red lines show individual patients treated in Arm 2. **(C)** Shown are CT images collected at baseline and 24 weeks (top panel) or 12 weeks (bottom panel) post-treatment for two individuals treated in Arm 1. Arrows point to lymph node metastases. **(D)** Percent changes in serum PSA values were evaluated from day of beginning pembrolizumab treatment (day 1 for patients in Arm 1, and week 12 for patients in Arm 2). **(E)** Best % change in serum PSA from day 1 of study. Asterisks indicate those patients who had evidence of PAP-specific Th1 immunity (significant IFNγ and/or granzyme B response detected at least twice post-treatment, Figure 2).

PCWG2 criteria were used for clinical and radiographic evaluation. As shown in Figure 3A, there was no difference in time to progression between study arms (HR=2.3, 95% CI: 0.8-6.9), and the overall median time to progression was 5.7 months (range 1.0-11.7+ months). Of the patients with measurable disease, 4/5 (80%) in Arm 1 and 1/3 (33%) in Arm 2 experienced any reduction in tumor volume, none meeting criteria for confirmed PR. Serum PSA declines from baseline were detected in 8/13 patients in Arm 1 compared to 1/12 patients in Arm 2 (p=0.01). Of PSA declines detected in Arm 1, one patient had a complete PSA response, and 3 additional patients had declines >25% (Figure 3B). Two of the patients with the greatest PSA declines also had reductions in tumor volume detectable by CT imaging (Figure 3C). Given that patients in Arm 2 received pembrolizumab beginning at 12 weeks, we also evaluated changes in PSA as a function of when they received pembrolizumab. As shown in Figure 3D, changes in serum PSA were only detected with concurrent treatment with vaccine. As further depicted by the asterisks in Figure 3E, PSA declines in patients treated in Arm 1 were associated (p=0.05) with the development of persistent T-cell immunity to the PAP target antigen (Figure 2A).

### Tissue studies

**Figure 4:**
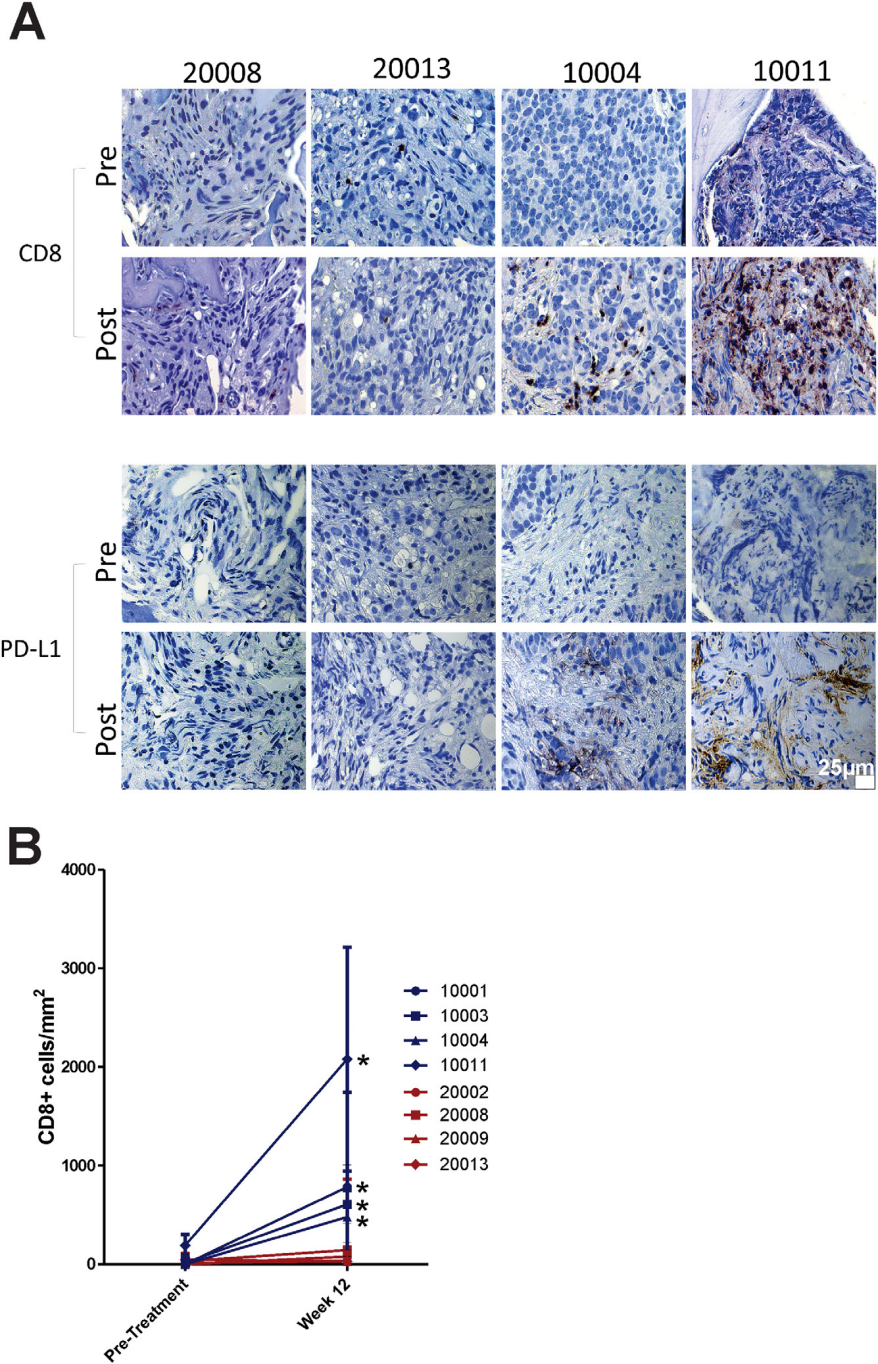
Tissue and correlative studies **(A)** Metastatic tissue biopsies obtained pre-treatment and at 12 weeks were evaluated by immunohistochemistry for CD8+ T cells and PD-L1 expression. Shown are representative sections from four individuals, two from each study treatment arm. 25μm ruler is shown in bottom right panel. **(B)** CD8+ T cell numbers in pre-treatment and post-treatment biopsy specimens were quantified by investigator blinded to treatment. Shown are the mean and standard deviation of CD8+ cell counts per mm^2^ from five regions per tumor section.

Tissue biopsies of metastatic lesions were obtained from 13 patients prior to treatment, 11 of whom had repeat biopsies of the same lesions at 12 weeks. These biopsies effectively sampled patients who had received vaccine only (Arm 2), or vaccine with pembrolizumab (Arm 1). Of these, eight had tumor present in both specimens suitable for analysis. Tissues were stained for the presence of CD8+ T cells and for PD-L1 expression. As shown in Figure 4A, CD8+ T-cell infiltration was detectable and statistically greater post-treatment in patients treated in Arm 1 (notably patients with serum PSA declines), but not Arm 2. Similarly, PD-L1 expression was induced, detectable post-treatment, in patients treated in Arm 1, but not Arm 2 (Figure 4B). Pre-treatment samples from several of these patients (10004, 10005, 10011, 20008, and 20009) were evaluated for microsatellite instability by staining for mismatch repair proteins MLH1, PMS2, MSH2, and MSH6; no evidence for microsatellite instability was found (data not shown).

## DISCUSSION

Immune based therapies have demonstrated remarkable clinical effects, leading *Science* to name cancer immunotherapy as the scientific breakthrough of the year for 2013 [20]. A great part of this success has been due to T-cell checkpoint inhibitors, including antibodies targeting PD-1/PD-L1 or CTLA-4. Notwithstanding, previous evaluation of these checkpoint inhibitors as monotherapies in advanced prostate cancer has demonstrated little benefit [13, 21, 22]. A possible exception may be advanced tumors with defects in DNA repair genes or rare subtypes of “inflamed” prostate tumors that have higher numbers of infiltrating T cells and/or PD-L1 expression on tumor cells [11]. Conversely, sipuleucel-T, an autologous cellular vaccine that targets the PAP prostate cancer antigen, and which acts presumably as a T-cell activating therapy, was demonstrated to lead to improved overall survival in patients with advanced prostate cancer despite few objective responses. The current trial sought to determine if combining anti-tumor vaccination with PD-1 blockade might be synergistic. This was based on preclinical studies demonstrating that the anti-tumor efficacy of DNA vaccines could be increased with PD-1 blockade employed at the time of PD-1 upregulation that occurs with vaccine-mediated T-cell activation [17, 18], and that PD-1 regulated T-cell immunity occurred in patients previously treated with a DNA vaccine encoding PAP [19]. To our knowledge, this is one of the first reports of a clinical trial using an anti-tumor vaccine in combination with PD-1 blockade, and the first report using a DNA vaccine. Our results demonstrate that this approach can yield objective tumor responses, an elusive endpoint for anti-tumor vaccines in advanced prostate cancers to date, and thus could potentially be explored for other tumor types that have demonstrated little effect from PD-1 blockade alone.

We wanted to investigate if it might be advantageous to block PD-1 expression that occurs at the time of initial T-cell activation with vaccination, and potentially not permit the activated cells to become dysfunctional within a PD-L1-expressing tumor microenvironment [18, 23]. We have previously demonstrated in mice that PD-L1 expression increases on tumors following immunization, and that PD-L1 expression increases on circulating prostate cancer cells shortly after vaccination in patients with prostate cancer who developed PAP-specific IFNγ-secreting T cells [17, 19]. Our results demonstrate that while vaccination elicited PAP-specific T cells in patients treated in either study arm, it was only when patients received concurrent PD-1 blockade that these cells demonstrated anti-tumor activity and CD8+ T-cell infiltration of metastases. Based on our previous studies, it seems likely that the infiltration of CD8+ T cells secreting IFNγ induced the expression of PD-L1 detectable after treatment in these individuals. Hence, contrary to the common perception of PD-L1 expression as a biomarker of response to PD-1 blockade, PD-L1 expression is a biomarker of IFNγ-secreting tumor-reactive T cells in the tumor environment on which PD-1 blockade may act. These findings suggest that vaccination may be a general means to increase tumor-reactive CD8+ T cells, permitting PD-1 blockade to work for patients with immunologically “cold” tumors, like prostate cancer, with low mutational burdens and generally low numbers of infiltrating lymphocytes.

As demonstrated in Figure 3, objective responses and PSA declines were generally associated with the development of IFNγ-secreting PAP-specific immune responses in patients treated with concurrent PD-1 blockade. Unfortunately, most PSA declines and radiographic changes reversed at the time treatment was stopped at 12 weeks. These observations suggest that the mechanism of anti-tumor response was specifically related to the development of immune response from vaccination and not, for example, due to defects in DNA repair as have been previously associated with response to PD-1 blockade [11]. As noted, we did not detect defects in DNA mismatch repair in patients analyzed. These findings have implications for future clinical trial designs. First, given that PSA declines were limited to subjects who received concurrent treatment, and generally limited to the period of treatment, the current trial was closed early prior to reaching the planned accrual goal. An expansion of the trial with continuous concurrent treatment beyond 12 weeks is currently being conducted. Second, the finding that objective clinical changes were associated with the development of immunity to the vaccine antigen suggest that efforts to increase the breadth of immune response to vaccination should yield better outcomes. We anticipate evaluating this approach using DNA vaccines targeting more than one antigen, ideally increasing the breadth of immunity to multiple tumor target antigens. Third, the finding that anti-tumor responses can occur using PD-1 blockade and a vaccine targeting a “shared” tumor antigen suggest that it may not be necessary to identify mutation-associated neoantigens as vaccine antigens. While this is a common contemporary approach, many cancers have low numbers of tumor-specific mutations, and the identification of tumor-specific neoantigens may not be feasible. Targeting shared antigens with T-cell activating vaccines for common cancers such as prostate cancer and breast cancer, or other cancers with low mutation burdens, could expand the therapeutic potential of PD-1 blockade, as these diseases are not typically responsive to PD-1 blockade alone. Finally, given the safety of the combination therapy, and the fact that objective changes were observed independent of androgen signaling pathways, we anticipate exploring this treatment approach in earlier stages of prostate cancer.

In summary, our results demonstrate that antigen-specific T cells were elicited following vaccination of patients with mCRPC using a DNA vaccine. When combined with concurrent PD-1 blockade, patients with evidence of Th1 immunity experienced PSA declines, objective tumor responses, and CD8+ T cell infiltration into metastatic lesions. These results suggest that the anti-tumor effect from vaccination can be augmented by concurrent delivery with PD-1 blockade and suggest a means, using tumor vaccination, to enable PD-1 blockade in immunologically “cold” tumors, like prostate cancer.

## MATERIALS AND METHODS

### Study agent and regulatory information

pTVG-HP is a plasmid DNA encoding the full-length human PAP cDNA downstream of a eukaryotic promoter [24]. The study protocol was reviewed and approved by all local and federal (FDA, NIH Recombinant DNA Advisory Committee) entities. All patients gave written informed IRB-approved consent for participation.

### Patient population

Subjects were patients with a histological diagnosis of prostate adenocarcinoma with metastases and castration resistance. Patients were required to continue androgen deprivation (surgical castration or GnRH analogue or antagonist treatment), and were required to have progressive disease, defined by consecutive rise in serum PSA, and/or increase in disease burden by CT or bone scintigraphy. Prior treatment with abiraterone and/or enzalutamide was allowed, however treatment with cytotoxic chemotherapy within 6 months of registration was prohibited. Prior treatment with sipuleucel-T was prohibited. Inclusion criteria required that patients have an ECOG performance score of < 2, and normal bone marrow, liver and renal function.

### Study design and procedures

This study was an open-label, single institution (University of Wisconsin Carbone Cancer Center), randomized pilot trial. The total accrual goal was 32 subjects, based on the goal of detecting an anticipated 45% increase in progression-free survival rate at 6 months in the concurrent treatment with 80% power at the one-sided 10% significance level. The trial schema is shown in Figure 1. Patients were treated six times at 14-day intervals with pTVG-HP plasmid co-administered with 200 mcg GM-CSF (Leukine®, sargramostim). Vaccinations were delivered intradermally with a 28-gauge needle on the lateral arm in two divided injections. Patients treated in Arm 1 received four doses of pembrolizumab (2 mg/kg administered intravenously) at 3-week intervals, beginning on the first day of immunization, over the first 12 weeks of treatment. Patients treated in Arm 2 received four doses of pembrolizumab (2 mg/kg administered intravenously) at 3-week intervals, but beginning 2 weeks after the last immunization, over weeks 12-24 of treatment. All patients underwent blood draws within two weeks of the first immunization, and at weeks 6, 12, 36 and 48, for immunological assessments. Patients also received a tetanus immunization prior to study treatment as an immunological positive control. Blood tests were performed approximately every 3 weeks and included CBC, creatinine, electrolytes, glucose, bilirubin, ALT, AST, alkaline phosphatase, amylase, LDH, and TSH. All toxicities were graded according to the NCI Common Terminology Criteria Grading System, version 4. Stopping rules were included to halt accrual if toxicity boundaries (35% > grade 3 or 20% grade 4) were exceeded.

### Clinical response evaluation

Serum PSA values were collected every 3-6 weeks. CT of abdomen/pelvis and bone scans were obtained within 6 weeks prior to the first day of treatment, and then at 12-week intervals following day 1. Tumor response measurements were made as per Prostate Cancer Working Group 2 (PCWG2) recommendations [25].

### Immunological response evaluation

Measures of antigen-specific immune response were performed by IFNγ and granzyme B ELISPOT with fresh (not cryopreserved) peripheral blood mononuclear cells (PBMC) as previously described [15, 16]. Antigens used included tetanus toxoid protein (Calbiochem), PAP protein (Research Diagnostics Inc.), or pools of 15-mer peptides spanning the amino acid sequence of PAP or PSA and overlapping by 11 amino acids (LifeTein, LLC). For these analyses, all antigens and sera used were from the same lots to control for possible variation over time. A response resulting from immunization was defined as a PAP-specific response detectable post-treatment that was statistically significant (compared to media only control by t-test), at least 3-fold higher than the pre-treatment value, and with a frequency > 1:100,000 PBMC, as we have previously reported [15].

### Tissue biopsy evaluation

Biopsies were obtained from a subset of subjects within 2 weeks prior to start of study treatment, and at week 12, from the same metastatic lesion. Tissue sections were stained immunohistochemically for expression of CD8 (CRM 311 A) and PD-L1 (ACI 3171 A) using antibodies purchased from BioCare Medical (Pacheco, CA) as per manufacturer’s specification. CD8+ cells were enumerated from five randomly selected 250 μm^2^ areas with mean counts +/- standard deviation reported.

### Statistical analysis

Outcomes and immunological parameters were summarized using descriptive statistics in terms of means, standard deviations, medians and ranges or frequencies and percentages. Two-sample t tests were used to compare antigen-specific T-cell response to media control at the two-sided 0.05 significance level. Time to radiographic progression was analyzed using the Kaplan-Meier method and compared between arms using the log-rank test. The frequencies and percentages of immunological responses were reported along with the corresponding 95% confidence intervals which were calculated using the Wilson-score method. Fisher’s exact test was used to compare immunological response rates between groups. The association between PAP-specific Th1 immunity and PSA changes was evaluated using a generalized linear model. Statistical analysis was conducted using SAS software (SAS Institute Inc., Cary NC), version 9.4.
